# Generation and characterization of monoclonal antibodies that recognize human and murine supervillin protein isoforms

**DOI:** 10.1371/journal.pone.0205910

**Published:** 2018-10-17

**Authors:** Tara C. Smith, Richard G. Saul, Elisabeth R. Barton, Elizabeth J. Luna

**Affiliations:** 1 Department of Radiology, Division of Cell Biology & Imaging, University of Massachusetts Medical School, Worcester, MA, United States of America; 2 Antibody Characterization Laboratory, Cancer Research Technology Program, Frederick National Laboratory for Cancer Research ATRF, Frederick, MD, United States of America; 3 Applied Physiology & Kinesiology, College of Health & Human Performance, University of Florida, Gainesville, FL, United States of America; Duke University School of Medicine, UNITED STATES

## Abstract

Supervillin isoforms have been implicated in cell proliferation, actin filament-based motile processes, vesicle trafficking, and signal transduction. However, an understanding of the roles of these proteins in cancer metastasis and physiological processes has been limited by the difficulty of obtaining specific antibodies against these highly conserved membrane-associated proteins. To facilitate research into the biological functions of supervillin, monoclonal antibodies were generated against the bacterially expressed human supervillin N-terminus. Two chimeric monoclonal antibodies with rabbit Fc domains (clones 1E2/CPTC-SVIL-1; 4A8/CPTC-SVIL-2) and two mouse monoclonal antibodies (clones 5A8/CPTC-SVIL-3; 5G3/CPTC-SVIL-4) were characterized with respect to their binding sites, affinities, and for efficacy in immunoblotting, immunoprecipitation, immunofluorescence microscopy and immunohistochemical staining. Two antibodies (1E2, 5G3) recognize a sequence found only in primate supervillins, whereas the other two antibodies (4A8, 5A8) are specific for a more broadly conserved conformational epitope(s). All antibodies function in immunoblotting, immunoprecipitation and in immunofluorescence microscopy under the fixation conditions identified here. We also show that the 5A8 antibody works on immunohistological sections. These antibodies should provide useful tools for the study of mammalian supervillins.

## Introduction

Cancer is the second leading cause of death in the United States and a major world-wide health problem [1]. Since the signing of the National Cancer Act in 1971, significant advances have been made in understanding how cancers arise and spread through the body, leading to novel therapeutic approaches and significant improvements in treatment outcomes [2]. The onset and metastatic spread of tumors involves the dysregulation of mechanotransduction, cell proliferation, growth factor signaling, vesicle trafficking and actin filament-based motile processes, including cell adhesion, translocation, cytokinesis and invasion of the extracellular matrix [3–8].

Isoforms of the membrane skeleton protein called supervillin are involved in each of these tumor-associated cellular processes [9–23]. Supervillin co-activates transcription with the androgen receptor [24–26], is up-regulated by estrogen [27], has been implicated in non-BRC1/2 breast cancer [28], and regulates the survival of U2OS bone osteosarcoma cells by regulating the deubiquitination and stability of p53 [14]. Supervillin also associates with the +8a isoform of the tumorigenic lysine-specific demethylase 1 (LSD1/KDM1A), an association that regulates neuronal maturation by controlling the methylation state of histone 3 at lysine 9 [29]. Although the role of supervillin in tumorigenesis is currently unclear [28, 30], these interactions suggest potential functions within the nucleus, as well as at the cytoplasm-membrane interface.

Supervillin protein isoforms arise from differential splicing of the 35 coding exons in the single human *SVIL* gene [12, 31, 32]. In nonmuscle cells, the most abundant splice-form is a ~205-kDa protein encoded by 31 exons [32–34]. This isoform was originally observed as an actin-binding protein on blot overlays and referred to as p205 [34, 35]; this protein is now called supervillin splice-form 1 or SV1 [14]. The largest supervillin isoform of ~250-kDa (p250), now called archvillin or SV2, contains sequence from all 35 *SVIL* coding exons [14, 31, 32]. With respect to supervillin, archvillin contains two additional sequences inserted into the supervillin N-terminus [31, 32]. Archvillin has so far been documented only in skeletal and cardiac muscle, but one or more of its 4 differentially spliced, “muscle-specific” *SVIL*-encoded exons are present in smooth muscle (SmAV or SV3) [15] and nonmuscle supervillin isoforms (SV4, SV5) [10, 14].

Investigations of the localizations within tissues and endogenous binding partners of the supervillin isoforms have been complicated by the difficulty of obtaining large amounts of specific antibodies. Mammalian supervillins are highly conserved, with consequently few antigenic sequences [31, 34, 36]. Affinity-purified rabbit polyclonal antibodies have been generated against bovine SV1 amino acids 900–918 [34], human SV1 residues 1–340 [33] and the large inserted sequence in human SV4 (residues 277–670) [37], but absolute specificity for *SVIL* gene products is hard to obtain and variable among rabbits and bleeds (see below).

As part of the National Cancer Institute’s Antibody Characterization Program, we report the generation and characterization of two murine and two rabbit monoclonal antibodies against human SV1 amino acids 1 through 340. As a group, these antibodies are appropriate for immunoblotting, immunoprecipitation, immunofluorescence and immunohistochemistry of human and murine supervillin isoforms. These antibodies, as well as plasmids encoding another three antibodies generated against the supervillin N-terminus, are being made available to the research community through the Developmental Studies Hybridoma Bank at the University of Iowa.

## Materials and methods

### Antigen preparation

The construction of the pGEX-6P-1 vector containing the coding sequence for the first 340 amino acids of human supervillin (h340) has been described [33]. A soluble GST-h340 fusion protein was obtained after expression in Rosetta2(DE3)pLysS bacteria (Novagen/EMD Millipore, Billerica, MA). Transformed bacteria were grown to late log-stage, induced overnight at 30°C with 0.2 mM isopropyl-β-D-1-thiogalactopyranoside, and purified on a column of glutathione-Sepharose 4B (GE Healthcare Life Sciences, Piscataway, NJ). GST alone was produced the same way to serve as a control in later assays. H340 protein was cleaved from GST-h340, using PreScission Protease (GE Healthcare Life Sciences) and dialyzed extensively against PBS (150 mM NaCl, 19 mM NaH_2_PO_4_, 8.1 mM Na_2_HPO_4_, pH 7.4). Trace amounts of uncleaved GST-h340 were removed with a second pass through glutathione-Sepharose 4B (GE Healthcare Life Sciences). H340 protein was sterilized by passage through a 0.22-μm syringe filter, adjusted to 1 mg/ml in PBS, and stored as frozen aliquots until used as an immunogen for the generation of polyclonal rabbit antisera and monoclonal murine hybridomas.

### Monoclonal antibody production

Mouse monoclonal antibodies were produced by Precision Antibody, Inc. (Columbia, MD) under the direction of the Antibody Characterization Lab (ACL) at Leidos Biomedical Research, Inc. (Frederick, MD). The ACL is funded by the National Cancer Institute’s Clinical Proteomic Technologies for Cancer initiative (CPTC) with the goal to provide reagents and other critical resources to support protein/peptide measurement and analysis efforts and thereby accelerate biomarker discovery and validation, cancer diagnostics development, and therapeutics monitoring. The detailed protocol (SOP) used by Precision Antibody to immunize mice and generate the monoclonal antibodies is available at http://antibodies.cancer.gov. Briefly, Swiss Webster derived mice (SJL/J strain; females 5–7 weeks old) were immunized with the target antigen (GST-h340) using a proprietary rapid, multiple immunization and hybridoma development platform. After 17 days, target-specific Ab titers were determined by ELISA (see below) against immobilized antigen by screening serial dilutions of the serum. Once a sufficient titer was detected, splenocytes were harvested from immunized mice with high serum titer to the target antigen and fused with a murine myeloma partner using a high efficiency electrofusion procedure. Direct cloning was performed in a semi-solid, hypoxanthine-aminopterin-thymidine selection medium prior to screening, utilizing an automated clone picking system. Supernatant from the resulting clonal hybridomas were subsequently screened by ELISA (see below) against the target antigen (h340, GST-h340) and a counter-screening antigen (GST). Positive clones with monoclonal antibodies specific to the target antigen were expanded and cryopreserved. IgG isotypes were determined using 1/8000 dilutions of ascites supernatants and Iso-Gold Rapid Mouse-Monoclonal Isotyping kits (Bioassay Works, LLC, Ijamsville, MD). Culture supernatants were further tested for specificity and utility in immunoblotting and immunofluorescence assays before final selection.

Clones 5A8 (CPTC-SVIL-3) and 5G3 (CPTC-SVIL-4) were grown in CELLine CL 1000 disposable bioreactor flasks (INTEGRA Biosciences, Zizers, Switzerland) at Precision Antibody, Inc. Secreted antibodies were purified by chromatography on affinity resins containing Protein A (5A8) or Protein G (5G3). Clone 5A8 was eluted with 50 mM glycine, pH ~3; clone 5G3 was eluted with 50 mM glycine, pH ~2.7. Both antibodies were neutralized by adding 1/10 volume of 1 M Tris, 1.5 M NaCl, pH 8.0. After sterile filtration, these antibodies were stable for weeks at 4°C. For longer storage, the antibodies were diluted with equal volumes of sterile glycerol and kept at -20°C, or stored undiluted at -80°C, with no obvious decline in efficacy.

### ELISA and IgG concentration determinations

The ELISA method entailed coating 100 ng/well of GST-h340 antigen in sodium bicarbonate buffer, pH 9.6, overnight at 4°C on high-binding, polystyrene microtiter plates. Wells were then rinsed with PBS and blocked for 1 h at room temperature with 3% milk, PBS. After washing plates 3 times with PBS, 0.05% Tween 20 (PBS-T), dilute mouse tail-bleed serum samples (1:1000, 1:3000, 1:10000, 1:30000 and 1:100000) in 3% milk, PBS were applied to the plate for 1 h at room temperature as the primary antibody. The plate was then washed thrice with PBS-T followed by the addition of secondary antibody (HRP goat anti-mouse IgG gamma chain specific) diluted 1:2000 and incubated for 1 h at room temperature. After washing plates 5 times with PBS-T, 3,3’,5,5’-tetramethylbenzidine substrate (100 μl) was added to each well and allowed to develop for 15–20 min at room temperature. Plates were read at 620 nm.

Antibody concentrations in hybridoma supernatants were estimated using bio-layer inferometry (BLI). Monoclonal antibodies were captured with an anti-mouse IgG Fc biosensor attached to the OctetR RED96 system (Pall ForteBio LLC, Fremont, CA). Signals were calibrated with a standard curve generated using total mouse IgG. Concentrations of purified antibodies were estimated from absorbance measurements at 280 nm, as determined with a Nanophotometer P300 spectrophotometer (Implen GmbH, Westlake Village, CA), and assuming an extinction coefficient of 1.4.

### Chimeric monoclonal antibody production

Hybridoma antibodies were sequenced from mouse hybridoma samples for clones 1E2, 2G3, 4A3, 4A8 and 5B8 using proprietary protocols. LakePharma (Belmont, CA) sequenced clones 1E2, 4A8 and 5B8; clones 2G3 and 4A3 were sequenced by Precision Antibody, Inc. PCR fragments containing the variable region sequences were used as templates for molecular construction. The variable regions for clones 1E2 (CPTC-SVIL-1) and 4A8 (CPTC-SVIL-2) were PCR amplified and cloned into pcDNA3.4 vectors, supplemented with rabbit IgG and rabbit kappa constant sequences, to generate the final expression constructs (LakePharma). Each DNA construct was scaled up for transfection and sequences were confirmed. A 0.1-liter transient production was carried out in HEK293 cells (Tuna293TM Process) for each antibody. The resulting monoclonal antibodies were purified by protein A affinity chromatography, eluted with a low pH buffer, filtered through a 0.2-μm filter and stored in 100 mM HEPES, 100 mM NaCl, 50 mM sodium acetate, pH 6.0.

### Epitope mapping

Epitope mapping was carried out by PEPperPRINT GmbH (Heidelberg, Germany), using a printed peptide microarray [38]. Each microarray contained 171 different peptides printed in duplicate, representing the complete sequence of the h340 immunogen, with GSGSGSG linkers at the N- and C-termini to avoid truncated peptides. Linear 15-residue h340 peptides with a peptide-peptide overlap of 13 amino acids were flanked by spots with a control HA peptide (YPYDVPDYAG, 60 spots). Microarrays were blocked for 30 min with blocking buffer MB-070 (Rockland Immunochemicals, Philadelphia, PA) and incubated preliminarily with secondary antibody (1:5000 goat anti-mouse IgG (H+L) DyLight680) to generate a secondary-only background that was used as a blank for the specific signal. Specific interactions were generated by shaking microarrays at 140 rpm for 16 h at 4°C with hybridoma supernatants diluted 1:10 and 1:1 in incubation buffer [90% PBS-T, 10% blocking buffer MB-070]. After washing, slides were incubated 45 min with 1:5000 goat anti-mouse IgG (H+L) DyLight680 in incubation buffer, and signals were visualized using a LI-COR Odyssey Imaging System (LI-COR Biosciences, Lincoln, NE), scanning offset 0.65 mm, resolution 21 μm, scanning intensities of 7/7. The HA peptides framing the peptide microarray were subsequently stained with 1:2000 mouse monoclonal anti-HA (12CA5) DyLight800 antibody as an internal quality control. Microarray image analysis was performed using PepSlide Analyzer (PEPperPRINT) and averaged spot intensities were calculated and plotted as a function of peptide localization in the h340 sequence. The CLUSTAL Omega multiple sequence alignment tool [39] was used to assess the relationships of the identified h340 epitopes and variable sequences with each other.

### K_D_ determinations using BLI

Kinetic properties were measured by BLI using a label-free ForteBio Octet RED96e instrument system (Pall Life Science, Port Washington, NY). All measurements were performed at 30°C in 96-well, black microplates (Greiner Bio-One, Monroe, NC; No. 655209) that were agitated at 1000 rpm. All protocols were performed according to the manufacturer’s instructions. Briefly, each antibody (20 μg/ ml) in 10 mM sodium acetate, pH 5, was covalently immobilized on amine-reactive second-generation sensors (AR2G; Pall ForteBio) via EDC/NHS coupling chemistry for 10 min. Excess reactive esters were blocked with 1 M ethanolamine, pH 8.5. After a brief acid conditioning with glycine buffer, pH 2.0, the antibody-coupled biosensors were neutralized in PBS-T. Kinetic measurements consisted of equilibration of the antibody-coupled biosensors in PBS-T for 600 seconds, followed by a baseline reading for an additional 600 seconds. The sensors were then dipped into wells containing the h340 antigen at concentrations of approximately 2, 4, 8 and 16 nM in PBS-T for 600 seconds to measure the association rate (on-rate). The sensors were then dipped into wells containing PBS-T for 600 seconds to measure the dissociation rate (off-rate). Rate constants for each antibody were calculated by applying a 2:1 interaction model (global fit, full) using the ForteBio data analysis software 7.0.1.5. Curves that could not be reliably fitted with the software (mostly full R^2^ < 0.96), were excluded from further analysis. Equilibrium dissociation constant (K_D_) values were calculated as the ratio of off-rate values to on-rate values.

### Cell culture, transfection and cell extracts

HeLa and U2OS cells were cultured using DMEM (high glucose, Life Technologies, Grand Island, NY) with 10% fetal bovine serum, sodium pyruvate, and 2.0 mM L-glutamine, in a humidified 37°C chamber with 5% CO_2_. Human rhabdomyosarcoma RH30 cells (American Type Culture Collection #CRL2061) were grown similarly in RPMI-1640 medium modified to contain 2 mM L-glutamine, 10 mM HEPES, 1 mM sodium pyruvate, 4500 mg/L glucose, 1.5 g/L sodium bicarbonate (American Type Culture Collection #30–2001), supplemented with 10% heat-inactivated fetal bovine serum. C57BL/6 murine embryonic fibroblasts (MEFs) were the generous gift of Dr. Stephen Jones (University of Massachusetts Medical School). Coverslips were coated with 5 μg/ml bovine fibronectin (Sigma-Aldrich Corp., St. Louis, MO) for ≥ 1 h before plating with U2OS cells. Transfection of EGFP-hSV1 [14], EGFP- or Flag-hSV4 [14] or EGFP-tagged murine archvillin (mAV, mSV2) [31] was carried out using Lipofectamine 2000 (Life Technologies), according to the manufacturer’s instructions. The transfection complexes were removed after 4 h. In knockdown experiments, we used Stealth dsRNAs (Life Technologies), as previously reported [18]. Briefly, the supervillin-specific dsRNA (SVKD) targeted hSV1 nucleotides 6016–6040 in the 3’-UTR: 5′-TATTAAGGTAGAAAGGTTGATT CGC-3′; the scrambled control sequence was 5′-GAACUAUGAAGGACCACCAGAGAUA. Plasmids encoding EGFP-tagged fragments of the bovine supervillin (bSV) N-terminus [19, 40] were transfected into RH-30 cells, as above. Cells were fixed or extracted 24 h after transfection with plasmids, or 48 h after transfection with dsRNAs.

Cell extracts were prepared as previously described [19]. Briefly, cells were rinsed with cold PBS, pH 7.4, then incubated on ice for 15 min in extraction buffer [150 mM sodium chloride, 50 mM Tris, pH 8.0, 1% Igepal CA 630, 0.5% deoxycholic acid, 0.1% SDS, 2 mM PMSF, and a protease inhibitor cocktail (#P8340, Sigma-Aldrich)] before collection and centrifugation at 15,000 x *g* for 15 min at 4°C. Extracts used for immunoprecipitation were made using extraction buffer lacking the 0.1% SDS.

### Other antibodies and stains

The affinity-purified polyclonal rabbit IgG used as a positive control was prepared and affinity purified on GST-h340, as described previously [31, 33]. A monoclonal mouse anti-actin (Clone C4, EMD-Millipore) was used to normalize supervillin signals in knockdown experiments. Monoclonal mouse anti-GFP (mix of clones 7.1 and 13.1), mouse anti-Flag (M2) and mouse anti-vinculin (hVIN-1) were from Sigma-Aldrich. Rabbit anti-Flag was from Cell Signaling Technology (Beverly, MA). Affinity-purified rabbit polyclonal anti-myosin IIA antibody (Poly19098, previously Covance Catalog #PRB-440P) and the non-specific mouse IgG2a were from BioLegend (San Diego, CA). Polyclonal rabbit anti-laminin was from ThermoFisher Scientific (FB-082-A). Goat anti-mouse or anti-rabbit HRP conjugated secondary antibodies used for chemiluminescent detection on immunoblots were from Jackson ImmunoResearch (West Grove, PA). Mouse monoclonal 5A8 was coupled to QDots-655 according to the manufacturer’s instructions (ThermoFisher Scientific, Waltham, MA). For immunofluorescence and immunohistochemistry, actin was visualized using Alexa Fluor-350 or -488 conjugated phalloidin (Life Technologies). Sarcolemmal costameres were stained with rabbit polyclonal antibody against the dystrophin C-terminus (ab15277, abcam, Cambridge, MA, USA). Secondary antibodies were goat-anti-mouse or anti-rabbit antibodies conjugated to either Alexa Fluor-488 or Alexa Fluor-568 (Life Technologies), as appropriate. Nuclei were stained using 4,6-diamidine-2-phenylindole dihydrochloride (DAPI, Sigma-Aldrich).

### Immunoblotting

Purified h340 immunogen and HeLa cell extract were resolved as curtain gels by SDS-PAGE (12% and 5–15% acrylamide, respectively) [41], transferred to 0.45-μm porosity Whatman Protran nitrocellulose (Whatman GmBH, Dassel, Germany), and cut into ~3 mm strips for immunoprobing with each hybridoma supernatant. Specificity for supervillin staining was tested using extracts from U2OS cells treated with either a supervillin-specific (SVKD) or control dsRNA resolved as adjacent lanes on 5–15% gradient gels, before transfer to nitrocellulose, as above. The replicate immunoblot strips contained molecular weight markers (RPN800E, GE Healthcare Life Sciences) and one lane each of SVKD and control extracts. Testing for immunoreactivity to murine supervillin was done on replicate blot strips of mouse embryonic fibroblast extract prepared as above. For immunoblotting, hybridoma supernatants were diluted 1:500 in blocking buffer (5% milk, 10 mM Tris-HCl, pH, 7.5, 166.5 mM NaCl, 0.05% Tween 20), and incubated with blot strips for 2 h at room temperature before secondary antibody probing. Affinity-purified polyclonal rabbit anti-supervillin h340 antibody was used at 1:5000 as a positive control and to evaluate the level of knockdown. Actin was used as a loading control to calculate percent knockdown [11]. Detection was by ECL [SuperSignal West Pico or Femto reagents (Thermo Scientific, Rockford, IL)], according to the manufacturer’s instructions, on a Chemidoc MP Imaging System, using ImageLab software, version 4.1 (Bio-Rad Life Science Research, Hercules, CA) [20].

### Immunoprecipitation

Cell extract was prepared, as above, from a single 10-cm dish of U2OS cells expressing EGFP-hSV1 for 24 h, and aliquots were divided equally among each antibody or control. Immunoprecipitations were carried out using protein A or G Dynabeads according to the manufacturer’s instructions (ThermoFisher Scientific). Briefly, 1 ml of hybridoma supernatant or control blank medium was mixed with 50 μl pre-washed protein G-coated Dynabeads for 1 hour with gentle rotation at room temperature. For IP of endogenous supervillin, 15 μg of antibody was mixed with 50 μl pre-washed protein A (rabbit IgGs) or protein G (mouse IgGs) on Dynabeads, as above. The beads were magnetically separated and the supernatant removed, then incubated in 200 μl of cell extract for 1 h at room temperature. After removal of the unbound supernatant, the beads were washed twice with half-strength TBST before being resolved on a 5%-15% SDS-PAGE gel, transferred to nitrocellulose, and probed with mouse anti-GFP or the chimeric rabbit 1E2 IgG. Development of the EGFP signal with HRP-conjugated anti-mouse antibody visualized both EGFP-hSV1 and the murine monoclonal heavy and light chains. Densitometric analyses were performed using ImageLab software (Bio-Rad). The percentages of immunoprecipitated EGFP-hSV1 were calculated by ratio to the amount of input EGFP-hSV1 and then normalized to the 5A8 clone via the immunoglobulin heavy chain signals. Percentages of bound endogenous supervillin were calculated by ratio of Unbound to Input signals.

### Immunofluorescence microscopy

Cells on coverslips were rinsed with room temperature PBS before fixation. For most experiments, fixation was with 4% paraformaldehyde (PFA) in CSK buffer (10 mM PIPES, pH 6.8, 300 mM sucrose, 100 mM sodium chloride, 3 mM magnesium chloride, 1 mM EGTA) [42] for 30 min on ice (CSK-PFA). The cells were then permeabilized at room temperature with 0.1% Triton X-100 in CSK buffer for 15 min. Other fixation methods tested included ice-cold methanol for 15 min (methanol), and 4% PFA in PBS for 10 min at room temperature [18]. Fixed cells were permeabilized with 0.1% Triton X-100 in CSK buffer for 4 min before incubation in blocking buffer (138 mM NaCl, 2.7 mM KCl, 8.1 mM Na_2_HPO_4_, 1.2 mM KH_2_PO_4_, 1% BSA, 0.5% Tween-20) for 30 min at room temperature. For staining, the hybridoma supernatants were diluted 1:5 in blocking buffer overnight at 4°C, or the purified monoclonal antibodies were used at 100 μg/ml for 2 h at room temperature. The coverslips were rinsed three times with blocking buffer before staining with appropriate secondary reagents and mounted onto slides with either SlowFade or ProLong Gold (Life Technologies).

Micrographs were obtained with a Leica DMI 6000B inverted fluorescence microscope with a 63X HC PL APO oil lens (N.A. 1.40) and a Leica DFC 365 FX camera, using Leica Application Suite 6000 software (Leica Microsystems, Exton, PA). Images were uniformly adjusted and assembled into figures using Adobe Photoshop (Adobe Systems Inc., San Jose, CA) software.

### Animals

Mice and rats were sacrificed by CO_2_ inhalation and cervical dislocation in accordance with procedures approved by the Institutional Animal Care and Use Committees at the University of Florida and the University of Massachusetts Medical School.

### Immunohistochemistry

Ten-micrometer frozen cross-sections from rat soleus muscle samples or murine extensor digitorum longus (EDL) muscles were fixed for 10 min with 4% PFA, washed and blocked, as described previously [21]. Sections to be labeled with unconjugated primary antibody were fixed as above, or in 4% PFA in CSK buffer on ice for 50 min, or in ice-cold methanol for 15 min, followed by permeabilization in 0.5% Triton X-100 in CSK buffer for 10 min. All tissue sections were incubated in immunofluorescence blocking buffer containing 5% normal goat serum (Jackson ImmunoResearch) for 30 min at room temperature. Mouse tissue sections were subjected to an additional blocking step with 0.1 mg/ml of unconjugated AffiniPure F(ab) fragment goat anti-mouse IgG (H+L) (Jackson ImmunoResearch) in blocking buffer for 1 h at room temperature. Primary antibodies were diluted to 0.1 mg/ml in blocking buffer with 5% goat serum, and QDot655-coupled clone 5A8 was diluted 1/100 before incubation for either 2 h at room temperature or overnight at 4°C. Slides were rinsed three times with the blocking buffer before incubation with the appropriate secondary reagents. Final rinsing was three times with PBS-T before mounting.

For confocal imaging, longitudinally sliced rat soleus muscle was stained as described above. Sarcolemmal costameres were imaged on a Leica SP5 (II) AOBS laser scanning confocal microscope (Leica Microsystems, Exton, PA) with an HCX PL APO CS 40.0X oil UV lens (N.A. 1.30), using Leica Confocal Software (Leica Microsystems). Thirty-three optical z-sections of 0.29 μm were obtained sequentially for each color channel through 10 μm, processed using maximum intensity projection and assembled using Adobe Photoshop.

## Results

Seven murine monoclonal antibodies specific for the first 340 amino acids in human supervillin (h340) were identified by ELISA, immunoblotting and immunoprecipitation. Hybridoma supernatants were first screened in an ELISA format to confirm that they reacted preferentially with the h340 immunogen, relative to a GST control (Table 1).

**Table 1:** ELISA screen of anti-supervillin monoclonal antibodies.

Clone #	h340	GST-h340	GST	[IgG] (μg/ml)	IgG isotype
1E2	> 3.000	> 3.000	2.208	14.7	IgG1
2G3	>3.000	>3.000	1.068	8.8	IgG1
4A3	2.64	2.544	0.61	13.6	IgG1
4A8	2.815	2.555	0.553	7.3	IgG1
5A8	2.939	> 3.000	1.063	14.7	IgG2a
5B8	>3.000	2.858	0.781	3.83	IgG2a
5G3	> 3.000	> 3.000	1.036	13.5	IgG1
NC	0.061	0.067	0.074	0.0	

NC: negative control.

**Fig 1:**
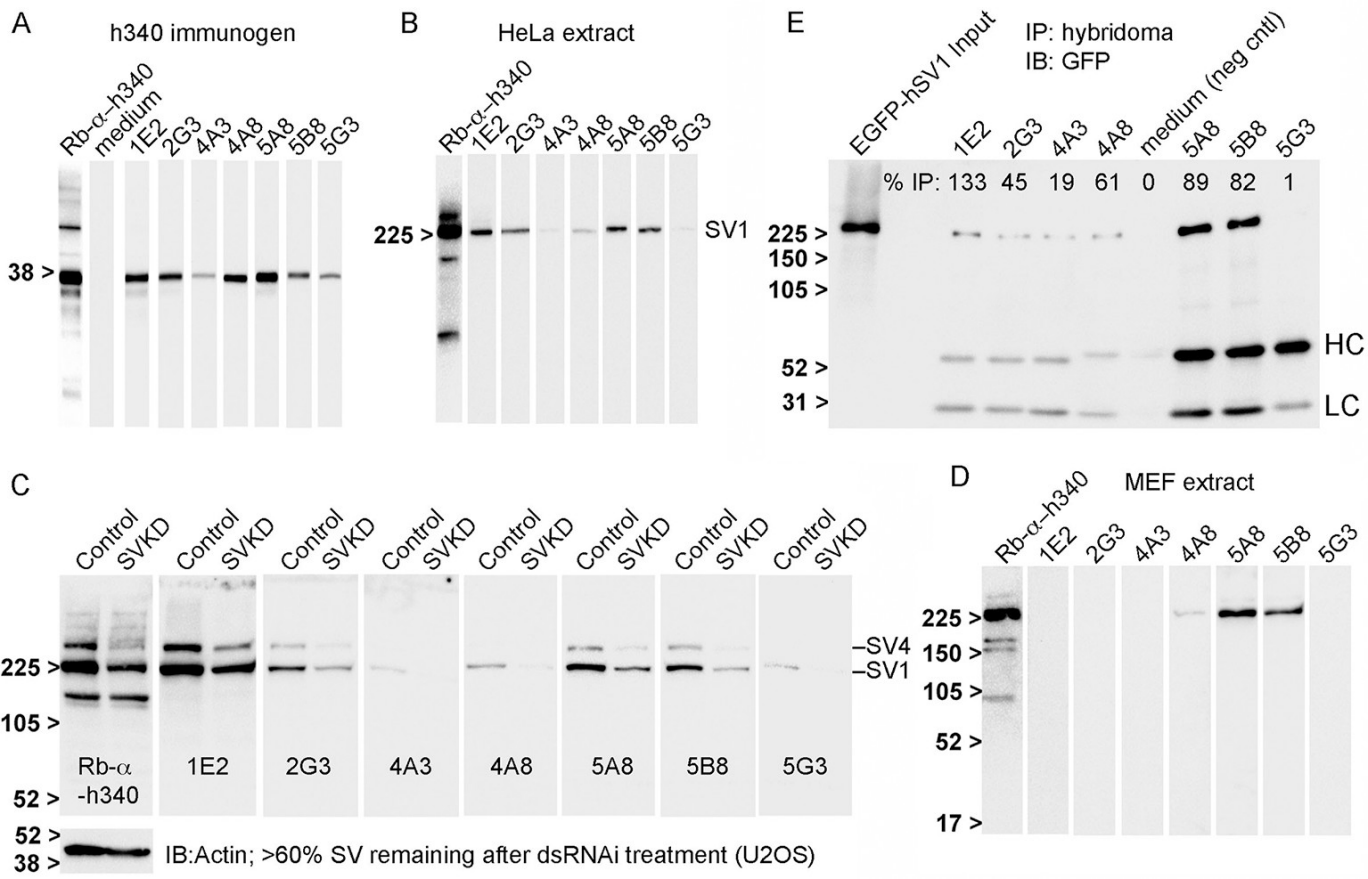
Monoclonal antibody reactivities in immunoblots and immunoprecipitations. Seven new monoclonal antibodies recognized human supervillin N-terminal sequences; three also recognized murine supervillin on immunoblots; six are efficacious for immunoprecipitation. Results of immunoblotting curtain-blot strips containing (A) the h340 immunogen resolved on a 12% SDS-gel, or (B) a HeLa whole cell lysate resolved on a 5–15% SDS-gel, with each of the new mouse monoclonal antibodies. Rabbit polyclonal anti-h340 was used as a positive control (Rb-α-h340); hybridoma medium as a negative control. Antibody reactivities are shown with equivalent exposure times after probing with equivalent dilutions of hybridoma supernatants. (C) Replicate blot strips were produced of cell extracts from human U2OS osteosarcoma cells transfected with either control (Control) or *SVIL*-specific (SVKD) dsRNA. The percentage of supervillin remaining after knockdown was 60%, based on probing with Rb-α-h340; total actin served as a loading control. (D) Curtain immunoblot strips of an extract from mouse embryonic fibroblasts (MEF extract), showing recognition of murine supervillin by clones 4A8, 5A8, and 5B8. (E) Co-immunoprecipitation of EGFP-hSV1 from a U2OS cell lysate by each monoclonal antibody coupled to protein G Dynabeads. Immunoprecipitated protein was visualized with mouse anti-GFP. An equal volume of hybridoma medium was the negative control (neg cntl). The percentages of immunoprecipitated EGFP-hSV1 were calculated from the ratio of normalized sedimented-to-input GFP signals, as described in Materials and Methods. HC, LC denote the locations of murine IgG heavy and light chains, respectively. Molecular weight markers in kDa, as indicated.

Efficacy in immunoblotting was then assessed using nitrocellulose strips containing either electrotransferred h340 immunogen (Fig 1A) or extracts from human cervical adenocarcinoma HeLa cells (Fig 1B), which contain primarily SV1 [14]. Ten of 16 tested hybridomas detected the ~38-kDa h340 polypeptide (Fig 1A), but only the 7 antibodies in Table 1 also recognized the endogenous ~205-kDa human SV1 (Fig 1B, SV1). The 3 antibodies that recognized only the h340 immunogen (not shown) were discarded without further testing. A recent preparation of affinity-purified rabbit anti-h340 antibody (Rb-α-h340) served as a positive control (Fig 1A–1D). The strongest signals were detected with the 1E2 and 5A8 clones, with moderate signals from clones 2G3, 5B8 and 4A8, and weaker signals from clones 4A3 and 5G3. There was no obvious correlation between immunoblot signal intensity and the amounts of IgG estimated by BLI (Table 1).

To confirm the specificities of these seven hybridoma supernatants, we examined their reactivities with human hSV1 and hSV4 in extracts from U2OS cells treated with either a well-documented supervillin-specific dsRNA (SVKD) or a scrambled control dsRNA in replicate immunoblot strips (Fig 1C) [14, 18]. Knockdown of supervillin to ~67% of the amount in control lysates was confirmed using the Rb-α-h340 antibody [33], with actin as a loading control. Both the SV4 and SV1 bands were reduced after treatment with the SVKD dsRNA, but the intensity of a ~160-kDa molecular mass band was unaffected (Fig 1C, first two lanes). Importantly, this nonspecific ~160-kDa band was not recognized by any of the 7 tested hybridoma supernatants (Fig 1C). Although their signals in control lanes varied, all 7 hybridoma supernatants showed reduced staining for both SV1 and SV4 in extracts from SVKD cells (Fig 1C).

Clones 5A8, 5B8 and to a lesser extent, 4A8 also recognized a high molecular mass supervillin isoform on replicate immunoblot strips of murine embryonic fibroblasts (MEFs) (Fig 1D). No signals were detected with the other 4 clones.

To confirm usefulness for immunoprecipitation, we tested the antibodies for their ability to recover EGFP-tagged hSV1 from a U2OS cell lysate (Fig 1E). Hybridoma medium alone was used as a negative control; the corresponding lane (medium) showed no sign of EGFP-hSV1 binding. All clones, except for 5G3, immunoprecipitated EGFP-hSV1 (Fig 1E). We noted that different amounts of murine IgG heavy (HC) and light (LC) chains were present in the immunoprecipitates, as revealed by the goat anti-mouse secondary antibody at ~50 kDa and ~25 kDa, respectively. Those amounts did not correspond with the calculated IgG amounts in Table 1 (1E2 and 5A8 should have equal amounts), suggesting variable affinities for the protein G beads. We thus calculated relative immunoprecipitation efficiencies from the ratio of bound EGFP-hSV1 to input EGFP-hSV1 (% IP, Fig 1E) and normalized to the amount of IgG heavy chain (HC), using 5A8 as the baseline. The results suggest that all clones, except 5G3, will precipitate EGFP-hSV1 and that clones 1E2, 5A8 and 5B8 are the most efficient.

**Fig 2:**
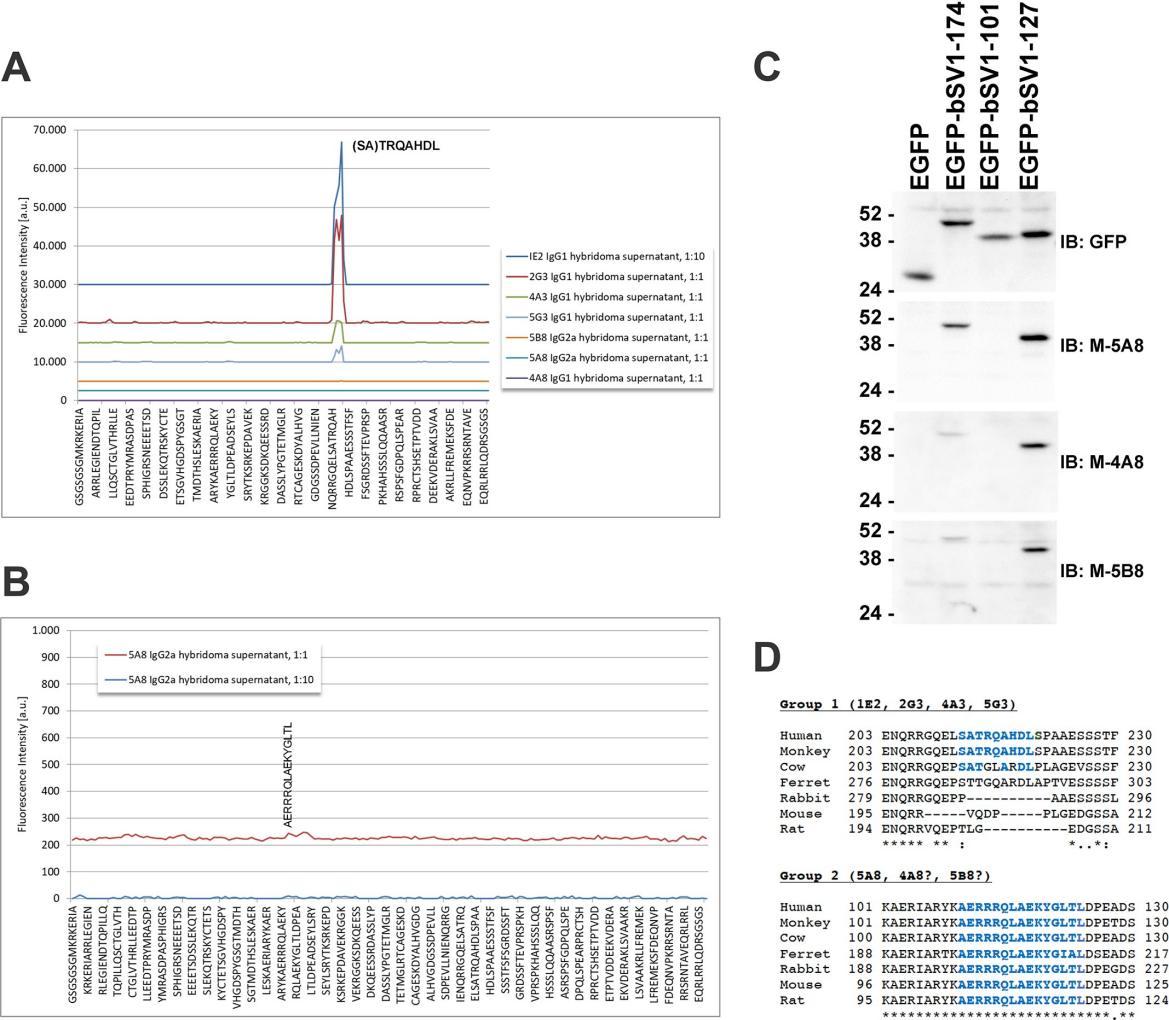
Epitope mapping. Seven monoclonal antibodies recognize two antigenic sequences in the supervillin N-terminus. (A) Superimposed representative intensity maps of signals from linear peptide microarrays showing that clones 1E2, 2G3, 4A3 and 5G3 recognize the consensus motif (SA)TRQAHDL, residues 215–223 in hSV1. Peptide overlap makes the requirement for the first two amino acids uncertain. (B) Amplification of the 5A8 intensity map suggests weak binding of clone 5A8 to AERRRQLAEKYGLTL, amino acids 110–124 in hSV1. (C) Recognition on immunoblots of overexpressed, truncated GFP-tagged bovine SV1 (bSV1) proteins in RH30 cell lysates shows that murine hybridoma supernatants (M) 5A8, 4A8 and 5B8 all recognize bSV1-174 and bSV1-127, but not bSV1-101 (D) Alignments showing the sequence conservation of the (SA)TRQAHDL and AERRRQLAEKYGLTL epitopes in mammalian supervillins. Species (reference sequence number): Human (NP_003165.2), African green monkey (ABA56494.1), cow (NP_776615.1), ferret (XP_012904085.1), rabbit (XP_008266240.1), mouse (NP_001334378.1), rat (NP_001101886.2).

The locations of the epitopes within the immunogen were interrogated by epitope mapping of each hybridoma supernatant with a printed microarray of 117 linear peptides [43, 44]. Clones 1E2, 2G3, 4A3 and 5G3 reacted with a range of avidities to overlapping peptides, including IENQRRGQELSATRQ and ELSATRQAHDLSPAA (Fig 2A). Clone 1E2 showed the highest avidity, followed by clone 2G3, and then clones 4A3 and 5G3. Thus, all these clones recognize a linear epitope; the predicted consensus motif is SATRQAHDL, which are residues 215–223 in hSV1. Clones 4A8 and 5B8 exhibited no detectable response to any of the tested linear peptides, whereas clone 5A8 reacted weakly with the single peptide AERRRQLAEKYGLTL, which corresponds to amino acids 110–124 in hSV1 (Fig 2B). These results suggest that the epitope(s) for clones 4A8, 5A8 and 5B8 are conformational, but may involve hSV1 residues 110–124. We tested this possibility by probing immunoblots of EGFP-tagged bovine supervillin (bSV1) truncation mutants [19] expressed in RH30 cells (Fig 2C). Anti-GFP recognized residues 1–174, 1–101 and 1–127 with nearly the same intensity (Fig 2C, top), but clones 5A8, 4A8 and 5B8 recognized only bSV1-127 and, to a lesser extent, bSV1-174 (Fig 2C). Thus, these 7 hybridomas fall into two groups (Fig 2D). Group 1 consists of clones that recognize the SATRQAHDL sequence, which is not well conserved among mammalian species, whereas Group 2 recognizes one or more conformational epitopes that require a highly conserved sequence within hSV1 amino acids 101–127 (Fig 2D).

The protein sequences of five of the hybridoma clones also fell into two groups (Table 2, S1 Fig). When the hybridoma clones, except for 5A8 and 5G3, were lost in a freezer failure, mRNA was extracted from vials of the lost clones, and the variable regions of the light and heavy chains were obtained by PCR amplification and DNA sequencing (Table 2). The variable heavy chain regions of the Group 1 clones 1E2, 2G3 and 4A3 were 91.4% identical, and the variable regions of their light chains were 60.2% identical. Furthermore, the lengths were identical, except for a 1-amino acid insertion in the 4A3 variable light chain sequence (S1 Fig). The variable sequences in 4A8 and 5B8 were divergent from the other clones and showed moderate similarity with each other. The 4A8 and 5B8 variable heavy and light chain regions were 64.2% and 55.0% identical, respectively, with a 9-amino acid insert in the 5B8 heavy chain variable region and a 5-residue insert in the 4A8 variable light chain region (S1 Fig). Two chimeric antibodies were then generated by cloning the 1E2 and 4A8 variable regions into pcDNA3.4 vectors containing sequences encoding rabbit IgG and rabbit kappa chain constant regions, co-expressing heavy and light chains in HEK293 cells and purifying each antibody on a protein A column. The resulting chimeric antibodies, one each from Group 1 and Group 2, are recognized by antibodies against rabbit IgG constant regions and used in parallel with purified murine monoclonal antibodies 5A8 and 5G3 in subsequent analyses.

**Table 2:** Accession numbers of sequenced clones.

		Accession	Numbers
Clone ID	Group	LC Variable Region	HC Variable Region
1E2	1	MH114969	MH114970
2G3	1	MH114971	MH114972
4A3	1	MH114973	MH114974
4A8	2	MH114975	MH114976
5B8	2	MH114977	MH114978

LC, light chain; HC, heavy chain. Immunoglobulin variable regions, partial CDS

**Fig 3:**
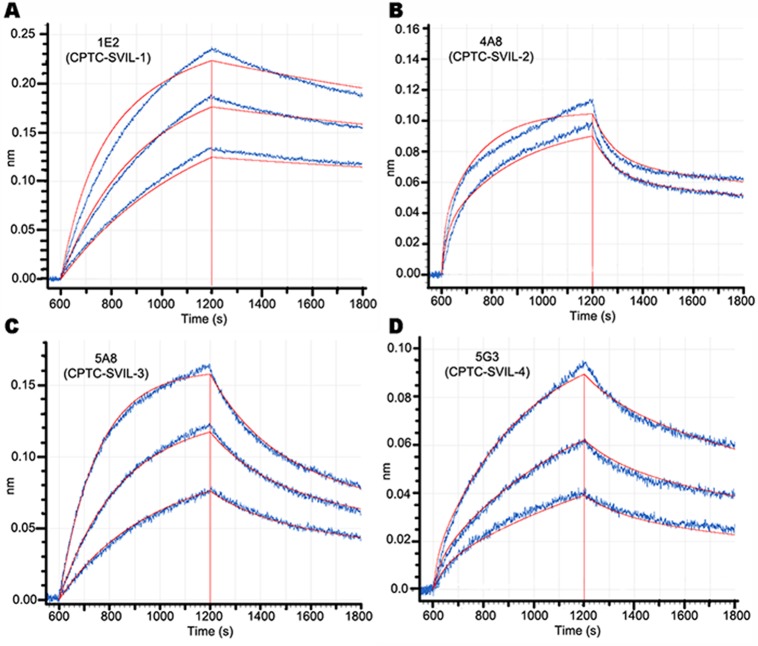
Kinetic binding curves. Binding to surface-bound h340 immunogen by (A) 4, 8 and 16 nM chimeric (rabbit Fc) monoclonal 1E2, R^2^ = 0.983, (B) 7.8 and 15.6 nM chimeric monoclonal 4A8 antibody, R^2^ = 0.965, (C) 1.95, 3.9 and 7.8 nM murine monoclonal 5A8, R^2^ = 0.997, and (D) 4, 8 and 16 nM murine monoclonal 5G3 antibody, R^2^ = 0.993. Calculated binding and dissociation curves are shown in red, as is the time of peak response. The calculated values for K_D_, k_ON_ and k_OFF_ are summarized in Table 3.

We used BLI association and disassociation curves to determine the kinetic binding properties of chimeric (rabbit Fc) 1E2 and 4A8 antibodies and murine monoclonal 5A8 and 5G3 antibodies (Fig 3, Table 3). Both chimeric antibodies (1E2, 4A8) bound to the h340 immunogen with nanomolar K_D_s, confirming that the correct antibody variable regions had been cloned in each case (Fig 3A and 3B). The highest peak responses were observed for antibodies 1E2 and 5A8, with ~4 nM of each generating responses of 0.1336 and 0.1218, respectively (Fig 3A and 3C). Antibodies 4A8 and 5A8 exhibited the tightest, subnanomolar binding constants (Fig 3B and 3C; Table 3). Surprisingly, given the differences in their relative reactivities on immunoblots and in immunoprecipitations (Fig 1), binding properties for h340 were similar for antibodies 1E2 and 5G3 (Fig 3A and 3D; Table 3). These subnanomolar and nanomolar K_D_s support the utility of each of these four antibodies for immunological applications.

**Table 3:** Binding constants to h340 of anti-supervillin antibodies.

Clone	Isotype	Epitope Group	K_D_ (nM)	K_ON_ (1/Ms)	K_OFF_ (1/s)
				(x 10^−5^)	(x 10^4^)
1E2	Rb IgG	1	4.21	1.18	4.96
4A8	Rb IgG	2	0.73	4.07	2.96
5A8	M IgG2a	2	0.53	7.28	3.87
5G3	M IgG1	1	3.22	1.50	4.85

Ms, moles/l • time (sec); Rb, rabbit IgG constant regions; M, mouse IgG constant regions.

**Fig 4:**
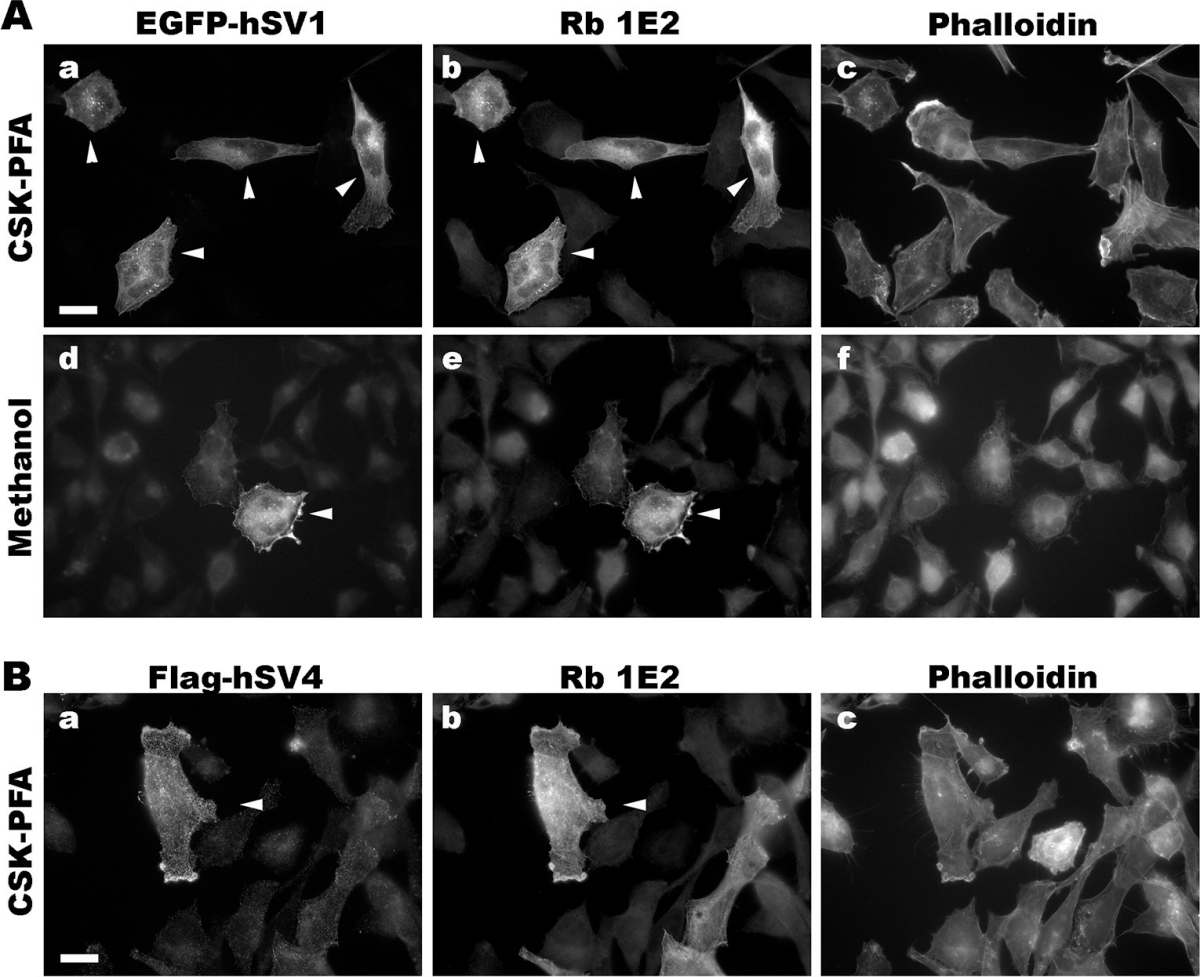
Representative immunofluorescence micrographs with chimeric rabbit 1E2 antibody (Rb 1E2). Co-localization of Rb 1E2 with (A) EGFP-hSV1 and (B) flag-hSV4 after overexpression in RH30 cells and fixation with either (a-c) CSK-PFA or (d-f) methanol. Cells transfected with the indicated supervillin isoform (panels a, d) were immunostained with the chimeric (rabbit Fc) monoclonal antibody 1E2 (panels b, e) and counterstained with phalloidin (panels c, f). Size bars, 20 μm. Note the close correspondence of the 1E2-staining with that of the EGFP or Flag tag on both hSV1 and hSV4 (arrowheads).

**Fig 5:**
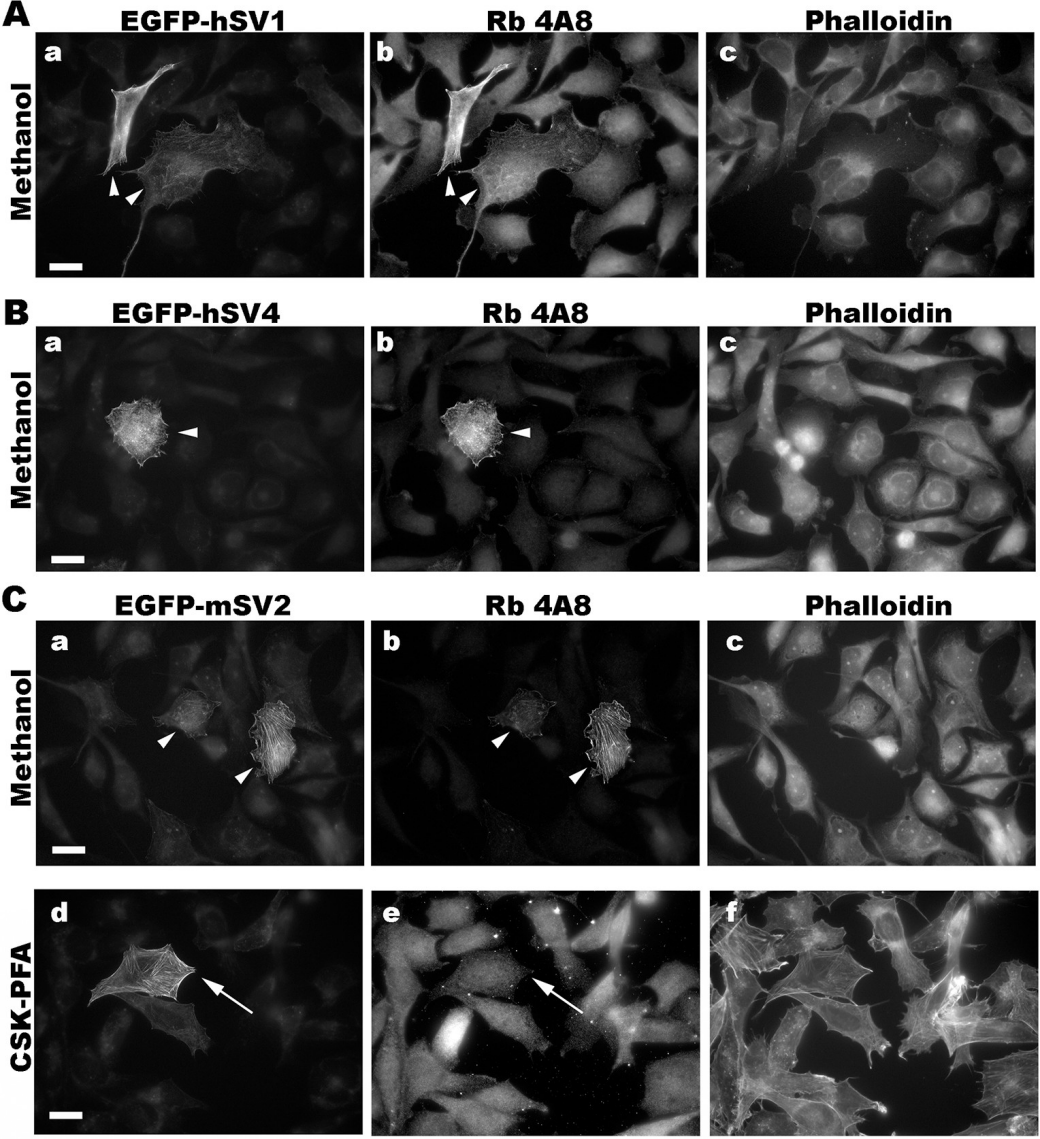
Representative immunofluorescence micrographs with chimeric rabbit 4A8 antibody (Rb 4A8). Co-localization of Rb 4A8 with (A) hSV1, (B) hSV4 and (C) mSV2 (murine archvillin) overexpressed in RH30 cells fixed with -20°C methanol. Cells transfected with an EGFP-tagged supervillin protein (panels a, d), as shown, were immunostained with the chimeric (rabbit Fc) monoclonal antibody 4A8 (panels b) and counterstained with phalloidin (panels c). Size bars, 20 μm. Note the close correspondence of the 4A8 staining with that of EGFP on each supervillin protein tested (arrowheads). After PFA fixation, little or no specific signal was observed (C, panels d-f, arrows).

**Fig 6:**
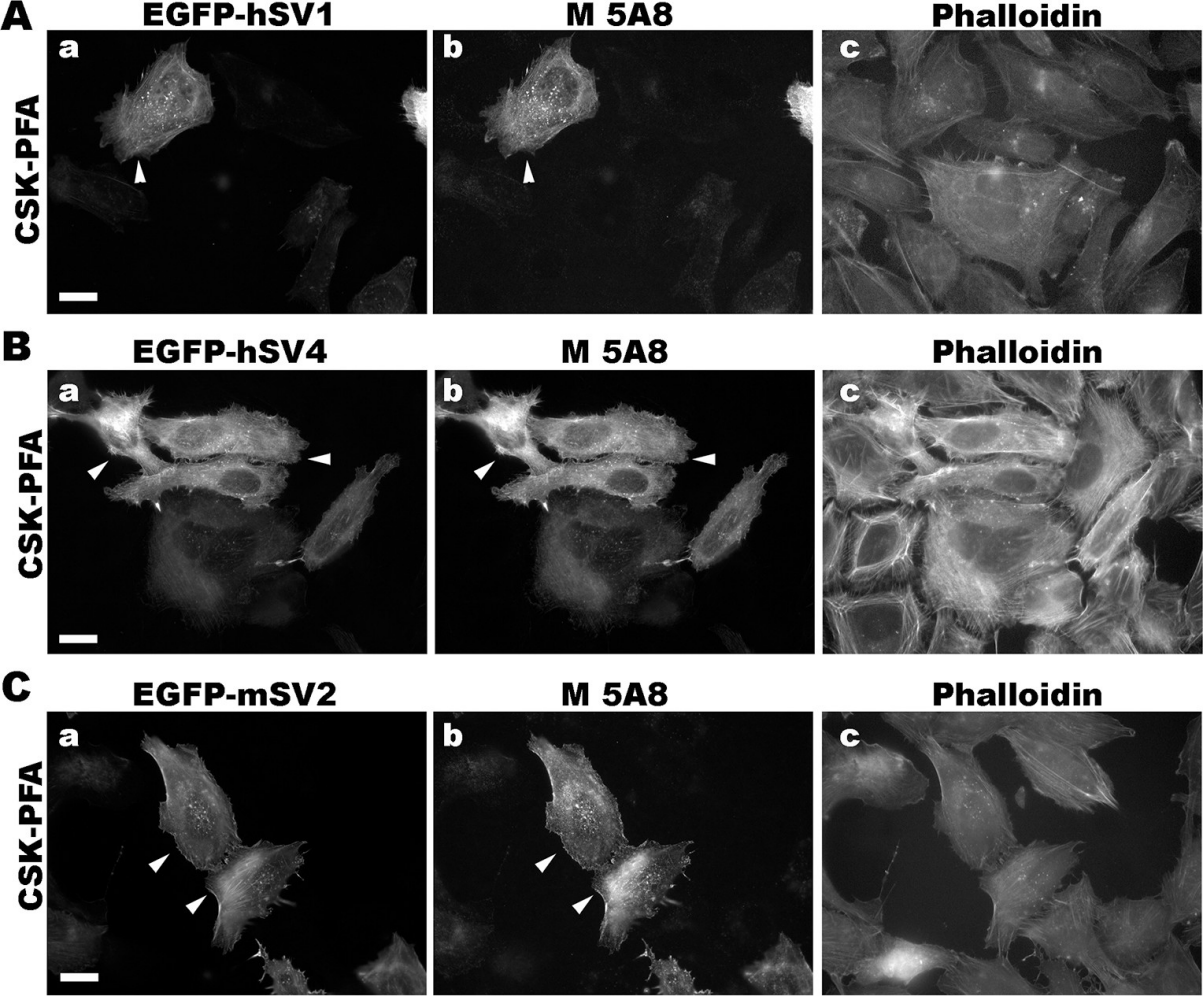
Representative immunofluorescence micrographs with murine monoclonal 5A8 antibody (M 5A8). Co-localization of M 5A8 with EGFP-tagged, overexpressed (A) hSV1, (B) hSV4 and (C) mSV2 (murine archvillin) in U2OS cells fixed with CSK-PFA. No specific signal was detected after fixation with -20°C methanol. Cells transfected with an EGFP-tagged supervillin protein (panels a), as noted, were immunostained with the 5A8 antibody (panels b) and counterstained with phalloidin (panels c). Size bars, 20 μm. Note the close correspondence of the 5A8-specific staining with that of EGFP on each supervillin protein tested (arrowheads).

**Fig 7:**
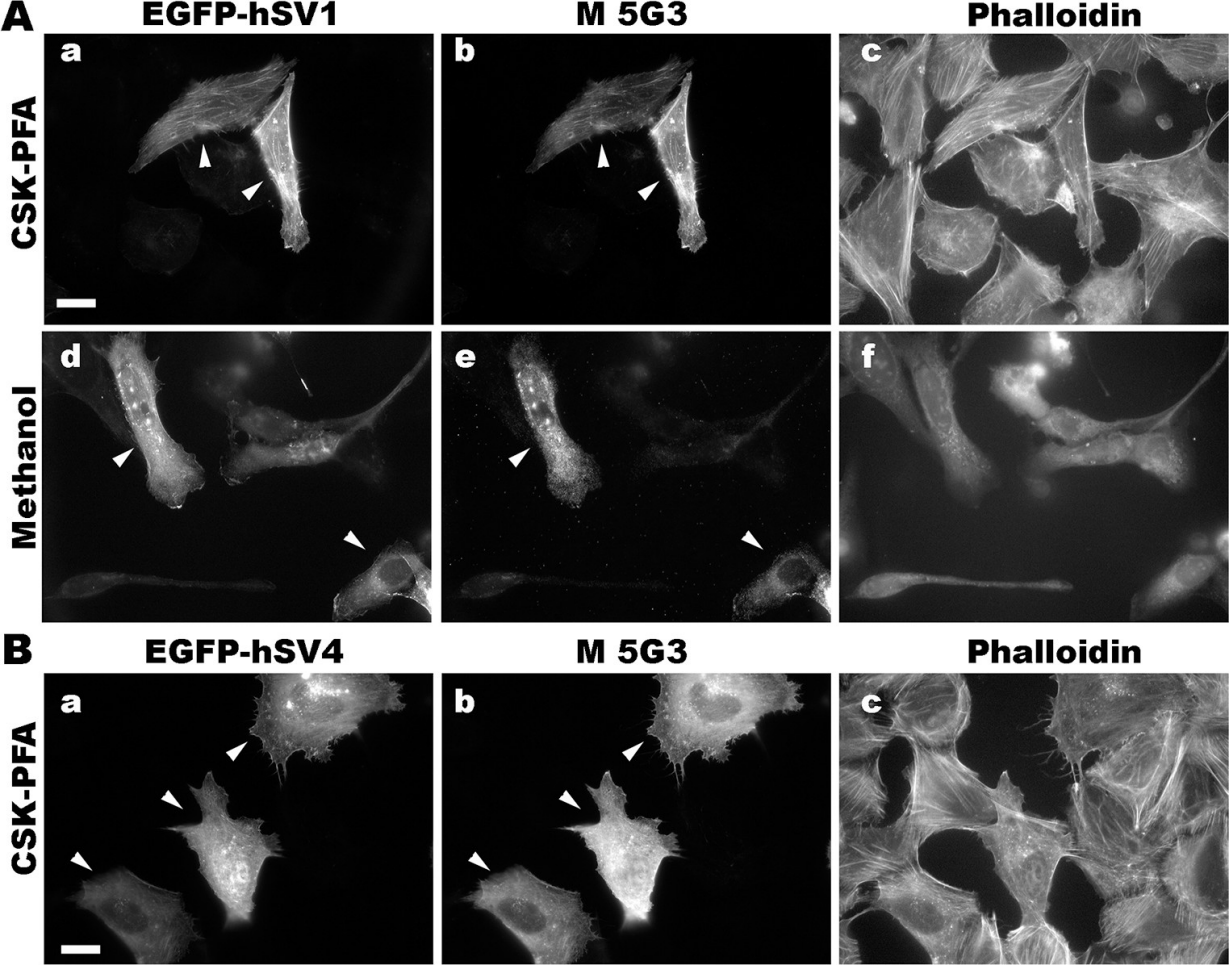
Representative immunofluorescence micrographs with murine monoclonal 5G3 antibody (M 5G3). Co-localization of M 5G3 with EGFP-tagged, overexpressed (A) hSV1 and (B) hSV4 in U2OS cells fixed with CSK-PFA, PBS-PFA or methanol, as indicated. The 5G3 antibody recognizes the Group 1 epitope, which is absent from non-primate supervillins. Cells expressing EGFP-hSV1 or EGFP-hSV4 (panels a, d) were immunostained with the murine monoclonal 5G3 antibody (panels b, e) and counterstained with phalloidin (panels c, f). Size bars, 20 μm. Note the close correspondence of the 5G3-specific staining (M 5G3) with that of EGFP on hSV1 and hSV4 (arrowheads).

We determined conditions for detecting the known isoforms of human and murine supervillins using an immunofluorescence microscopy screen and found that the new antibodies have distinct fixation requirements (Figs 4–7). To create cells with and without high concentrations of defined supervillin isoforms, we transfected U2OS osteosarcoma or RH30 rhabdomyosarcoma cells with plasmids encoding EGFP-hSV1, EGFP- or Flag-tagged hSV4, or GFP-mSV2 (murine supervillin, isoform 2; archvillin; MAV) [14, 31]. We used phalloidin staining as a marker for actin filaments as a reference for known supervillin localizations along stress fibers and at focal adhesions in PFA-stained cells and as a marker for cytoplasm after fixation with methanol. We looked for corresponding cell-to-cell differences in staining intensities and for the alignment of the EGFP or Flag reactivity with each anti-supervillin monoclonal antibody. We tested two cell-fixation methods: (1) -20°C methanol and (2) 4% PFA in either PBS or CSK buffer [42]. Images were obtained at optimal exposure times for each antibody. Sample images are shown in Figs 4 through 7, and the results from these screens and from experiments with endogenous supervillin are summarized in Table 4 below.

**Table 4:** Summary of antibody reactivities in immunofluorescence microscopy.

Clone	cPFA GFP-hSV1	Methanol GFP-hSV1	GFP/flag-hSV4	GFP-mSV2	Endogenous signal
Rb 1E2	Yes	Yes	Yes	No	Yes
Rb 4A8	Weak	Yes	Yes*	Yes*	Weak or No
M 5A8	Yes	No	Yes	Yes	Yes
M 5G3	Yes	Yes	Yes	n.d.	Weak or No

Rb, rabbit IgG constant regions; M, mouse constant regions; cPFA, paraformaldehyde fixation in CSK buffer; methanol, fixation with -20°C methanol

*, methanol-fix only; n.d., not determined (epitope not in mSV2).

We found that the chimeric 1E2 antibody, which recognizes Epitope 1, visualized both hSV1 and hSV4 under both fixation conditions (Fig 4). Recognition of hSV1 was strong after cells were fixed with either CSK-PFA (Fig 4A, a-c) or methanol (Fig 4A, d-f). The 1E2 signals co-localized almost perfectly with the EGFP fluorescence (Fig 4A, b and e *vs*. Fig 4A, a and d; arrowheads), suggesting specificity. Recognition of the 1E2 epitope at hSV1 amino acids 215–223 (Fig 2D) was unaffected by the insertion of the additional amino acid sequences after hSV1 residue 276 that characterize hSV4 and other supervillin splice-forms [10, 14, 15, 31] (Fig 4B). Thus, the chimeric 1E2 antibody should recognize Epitope 1 in all these splice-forms of human and primate supervillins.

The chimeric 4A8 antibody, which requires sequences around the highly conserved Epitope 2 for binding (Fig 2C and 2D), recognizes mSV2, as well as hSV1 and hSV4 (Fig 5), suggesting utility across mammalian supervillins. However, our experiments suggest that methanol fixation is preferable for this antibody (Fig 5A, 5B and 5Cb) because PFA fixation frequently resulted in a weak or nonspecific signal (Fig 5Ce).

The 5A8 mouse monoclonal antibody, which also recognizes Epitope 2 (Fig 2), predictably recognizes both human and murine supervillin proteins (Fig 6). In contrast to observations with the chimeric 4A8 antibody, the 5A8 antibody recognized supervillin only after PFA fixation (Fig 6); no immunofluorescence signals were observed with methanol-fixed cells (not shown). Thus, the 5A8 and 4A8 antibodies may be complementarily useful for immunofluorescence of murine supervillin isoforms, with epitopes differentially sensitive to fixation technique. Because 4A8 is recognized by anti-rabbit secondary antibodies, whereas 5A8 is a murine monoclonal (Table 3), these antibodies also provide the capability to co-localize mammalian supervillins with proteins recognized by either anti-mouse or anti-rabbit primary antibodies.

The mouse monoclonal 5G3 antibody, like the chimeric 1E2 antibody, recognizes Epitope 1, which means that its efficacy is limited to supervillins from humans or other primates (Fig 2). We find that, also like the chimeric 1E2 antibody (Fig 4), the 5G3 antibody recognizes both hSV1 and hSV4 after fixation with PFA in CSK buffer (CSK-PFA, Fig 7A, a-c; 7B, a-c), hSV1 fixed with PFA in PBS (PBS-PFA, Fig 7A, d-f), or hSV1 fixed with ice-cold methanol (Methanol, Fig 7A, g-i). Despite its poor showing in immunoblotting (Fig 1), the 5G3 antibody effectively recognized human supervillin isoforms in immunofluorescence and can be used for experiments involving primate supervillins when a murine secondary antibody is preferable.

**Fig 8:**
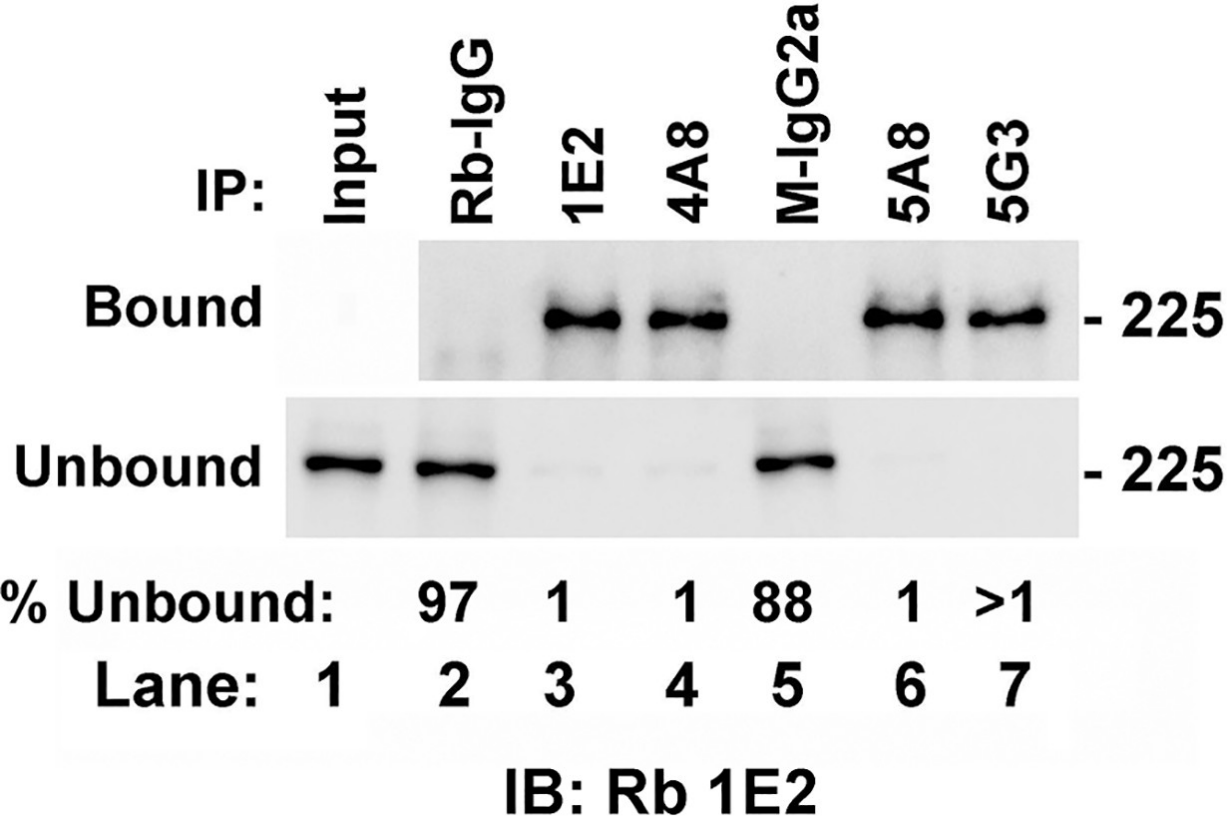
Each of the new monoclonal antibodies immunoprecipitates endogenous human supervillin. Bound and unbound supervillin from an RH30 cell lysate are shown after immunoprecipitations with the antibodies indicated at the top of the figure, SDS-PAGE and immunoblotting with Rb 1E2 antibody. Large amounts of supervillin (>90%) co-pelleted with each specific antibody in the bound fractions (top). Input (lane 1) is shown with the unbound fractions, all of which showed signal depletion after incubation with supervillin-specific (lanes 3, 4, 6, 7), but not with control (lanes 2, 5) antibodies. The percentage of the unbound intensity relative to Input is shown at the bottom of each lane. This blot is representative of two independent experiments.

To determine antibody efficacy with endogenous levels of supervillin isoforms, we first tested each antibody for the ability to immunoprecipitate endogenous protein from human RH30 cells (Fig 8). Antibodies 1E2, 4A8, 5A8 and 5G3 all efficiently immunoprecipitated endogenous supervillin, leaving little in the unbound fraction (Fig 8A, lanes 3, 4, 6, 7). By contrast, essentially no supervillin was recovered with either the rabbit IgG (Rb-IgG, Fig 8A, lane 2) or with murine IgG2a (Fig 8A, lane 5) used as negative controls.

**Fig 9:**
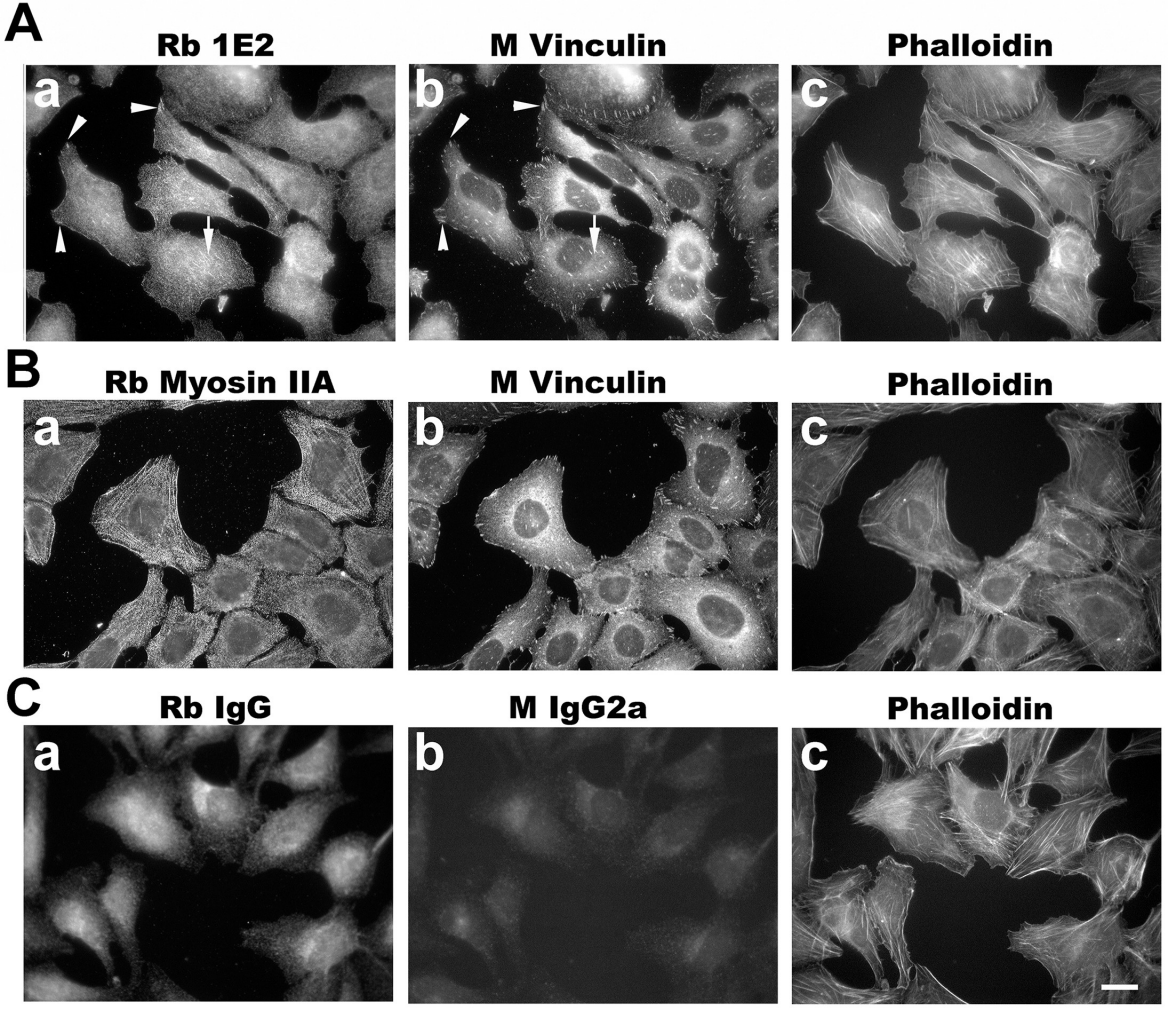
Representative immunofluorescence staining of endogenous human supervillin by chimeric Rb 1E2. (A) U2OS osteosarcoma cells cultured on fibronectin for 24 h were fixed with CSK-PFA and stained with the chimeric rabbit monoclonal antibody 1E2 (panel a) and monoclonal anti-vinculin (panel b). (B) Similarly-cultured U2OS cells were stained with rabbit polyclonal anti-myosin IIA antibody (panel a) to show that the linear elements seen in panel Aa are probably stress fibers (panel Aa, arrows), whereas staining with monoclonal anti-vinculin (panel b) shows the presence of focal adhesions (panel Ab, arrowheads) at the membrane-proximal ends of these actin filament bundles. Phalloidin staining (panels c) also visualized these supervillin-associated structures. (C) Neither stress fibers nor focal adhesions are stained with control rabbit IgG (panel a) or non-specific murine IgG2a (panel b). Size bar, 20 μm.

The best staining of endogenous supervillin in human U2OS cells by immunofluorescence microscopy was obtained with the chimeric 1E2 clone (Fig 9). Each antibody was tested for the ability to recognize endogenous supervillin at focal adhesions (marked by vinculin) and along stress fibers (marked by myosin IIA) in U2OS cells plated on fibronectin and fixed with CSK-PFA. We looked for co-localizations along actin filament bundles (Fig 9, panels c), which were likely stress fibers based on the myosin II signal in these cells (Fig 9Aa, arrow; Fig 9Ba), and for foci at the ends of these structures (Fig 9Aa, arrowheads) that are presumptive focal adhesions, based on vinculin signals [45, 46]. Both of these cellular structures are known to contain supervillin [23, 47]. Consistent with its colocalization with overexpressed hSV1 and hSV4 (Fig 4), 1E2 co-stained some vinculin foci (Fig 9Aa-b, Fig 9Bb, arrowheads) and localized along interior actin filaments associated with vinculin foci, presumably stress fibers (Fig 9Aa, arrow). These signals were not observed with either the rabbit or murine IgG controls (Fig 9Ca and 9Cb). Staining with the other three clones was not distinctive (not shown), but their efficacies with overexpressed supervillin isoforms suggests either a problem of antigen abundance or availability when incorporated into complex structures. These results suggest that while each antibody may be useful for immunostaining of endogenous supervillin in some experimental contexts, 1E2 is preferable for endogenous human supervillin, with 5A8 or 4A8 required for staining in non-primate species.

Table 4 summarizes the results from Figs 4 through 7 and Fig 9. We found that chimeric rabbit (Rb) 1E2 or mouse (M) monoclonal 5A8 were the best choices for most immunofluorescence experiments. Both are excellent reagents for human supervillin proteins; together, they provide flexibility in double-labeling experiments with other desired antibodies. Mouse monoclonal 5A8 is the best choice for PFA-fixed non-human samples. The chimeric rabbit 4A8 is the only antibody that will visualize non-primate supervillins after methanol fixation.

**Fig 10:**
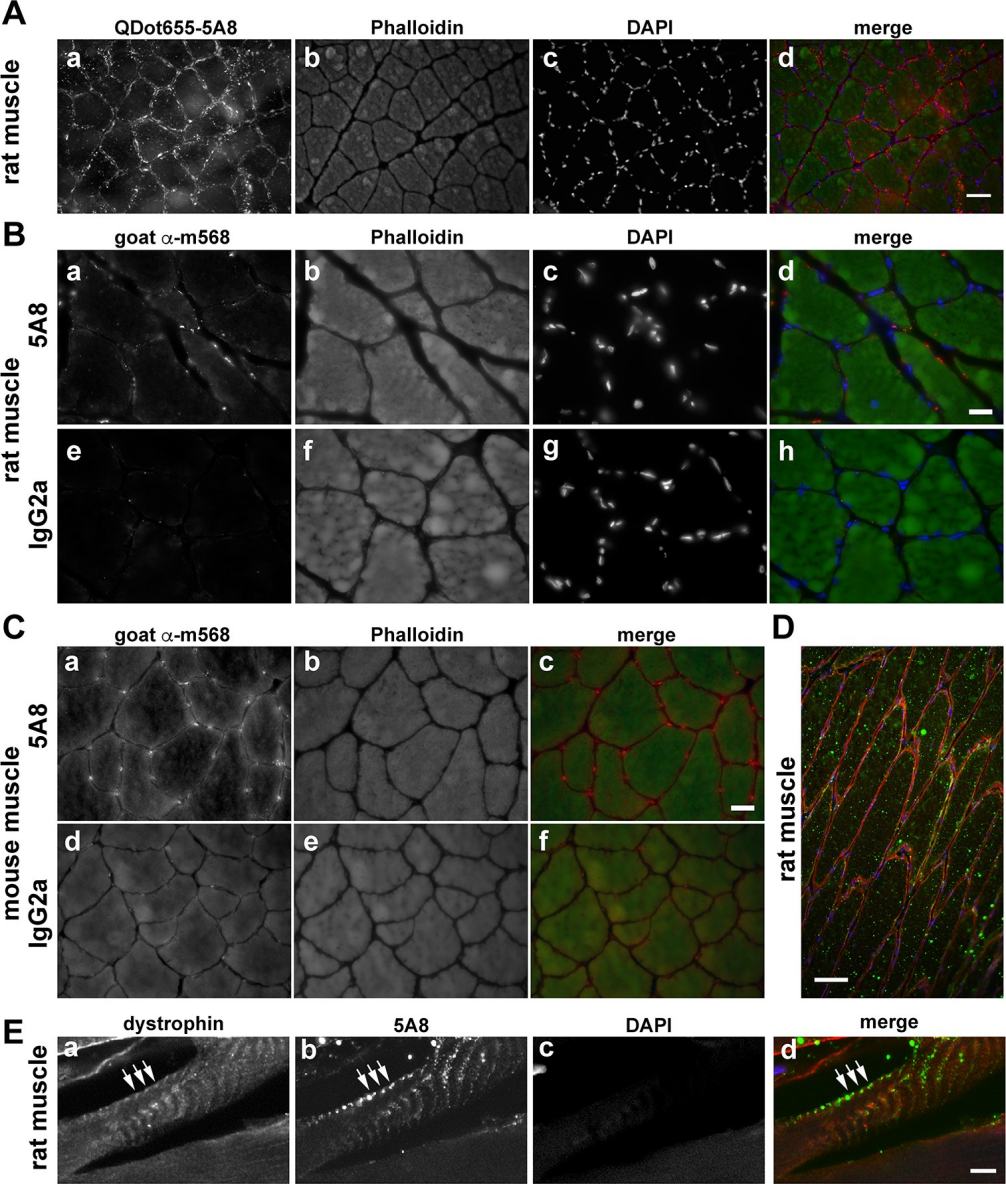
Immunohistochemical staining of rat and mouse muscle sections. Specific staining of archvillin in rat soleus and mouse EDL muscles with murine monoclonal antibody 5A8 (5A8). (A) Cross-sections of PFA-fixed rat soleus muscle were stained using QDot655 labeled 5A8 (a, red in merge), phalloidin (b, green in merge) and DAPI (c, blue in merge). Color merge shown in (d). Bar, 50 μm. (B) PFA-fixed rat soleus muscle probed with clone 5A8 (a) or with a non-specific mouse isotype-matched control IgG2a (e), both red in merge. The slide also was stained for phalloidin (b, f, green in merge) and DAPI (c, g, blue in merge). Merges are shown in panels d, h. Bar, 20 μm. (C) Mouse EDL muscle fixed and stained as in (B) with either the monoclonal 5A8 (a) or the IgG2a isotype control (d) and phalloidin (b, e, green in merge). Merges shown in panels c, f. Bar, 20 μm. (D, E) Unfixed rat soleus muscle after longitudinal cryo-sectioning and staining with anti-dystrophin (a, red in merge), 5A8 (b, green in merge), and DAPI (c, blue in merge) and visualization by wide-field immunofluorescence microscopy (D) or maximum point projection after confocal imaging (E). Merges shown in panels D and E. Bar in D, 50 μm; bar in E, 5 μm.

The last application tested was immunohistochemistry. Both murine-reactive clones (mouse 5A8 and rabbit 4A8) were tested on rat and mouse skeletal muscle cross-sections fixed with PFA (Fig 10). Skeletal muscle is known to be enriched in the SV2 / archvillin isoform mRNA and protein, as compared to other tissues, and the localization of archvillin with dystrophin at the sarcolemmal membrane is well documented [21, 31, 32]. In an attempt to avoid non-specific staining with secondary antibodies, the 5A8 antibody was labeled directly with Qdot655 (Fig 9A), and its localization examined in reference to nuclei and phalloidin staining. Sections showed intense puncta surrounding the muscle fibers at the sarcolemma. Because Qdot penetration into cells can be an issue [48], we also stained rat (Fig 10B) and mouse muscle sections (Fig 10C) with unlabeled 4A8 and 5A8 antibodies and compared the resulting signals with those from sections stained with non-specific primary antibodies of the same isotype. Unfortunately, the signal from the rabbit 4A8 clone was indistinguishable from the control antibody on muscle sections fixed with methanol (not shown). In contrast, the signal from the mouse 5A8 antibody (Fig 10B, 10C) was similar to that of the QDot655 labeled 5A8 (Fig 10A), which resembled a subset of the previously observed sarcolemmal staining [31]. Longitudinal sections of unfixed rat muscle showed both linear and punctate sarcolemmal staining with 5A8 (Fig 10D and 10E). This 5A8 staining co-localized with dystrophin at costameres (Fig 10E, arrows), supporting its effectiveness for immunohistochemical staining of supervillin isoforms.

## Discussion

We describe here the generation and characterization of four new monoclonal antibodies reactive with N-terminal sequences shared among supervillin isoforms. Two antibodies (1E2/CPTC-SVIL-1; 5G3/CPTC-SVIL-4) recognize a primate-only sequence in hSV1 amino acids 215–223, whereas the other two antibodies (4A8/CPTC-SVIL-2; 5A8-CPTC-SVIL-3) recognize an evolutionarily conserved conformational epitope that requires hSV1 residues 101 to 127. One antibody in each pair is a murine monoclonal, and the second is a chimeric monoclonal antibody recognized by anti-rabbit secondary reagents. All four antibodies work for immunoblotting and immunoprecipitation of endogenous supervillin. Three antibodies (1E2, 4A8, 5A8) are effective in immunofluorescence microscopy under conditions identified here, and the 5A8 antibody recognizes endogenous protein in immunohistological sections of mouse and rat muscle.

Especially in pairs, these antibodies will be useful for most investigations involving supervillin. The chimeric rabbit 1E2 antibody and the murine 5A8 antibody performed the best in experiments with human or other primate supervillin isoforms. Each antibody specifically recognizes one of the two identified antigenic sequences in the supervillin N-terminus, which is shared among supervillin isoforms, and each was efficacious in all tested assays with human supervillins. For murine or rat samples, chimeric rabbit 4A8 and murine 5A8 are the best choices. Each of these antibody pairs gives the investigator the choice of using an anti-rabbit or an anti-mouse secondary reagent, which can be useful for co-immunolocalization experiments with other antigens. Another consideration for immunofluorescence co-localizations is that the chimeric rabbit 4A8 antibody works most consistently after methanol fixation, whereas the murine 5A8 clone works best after PFA fixation in CSK buffer.

Although less effective than the other three antibodies in all tested assays, the murine 5G3 antibody does immunoprecipitate endogenous supervillin and recognize overexpressed human supervillin in immunofluorescence microscopy. The 5G3 binding affinity for the h340 antigen is comparable to that of the chimeric rabbit 1E2, so the reasons for the differences in efficacy in immunoblotting, immunofluorescence and immunoprecipitation, e.g. Fig 1E vs. Fig 8, are unclear. One possibility is that N-terminal EGFP or various supervillin interactors differentially block access to the 5G3 epitope. Nevertheless, the mouse monoclonal 5G3 clone could be useful in experiments that require two different anti-supervillin antibodies, such as for immunoprecipitation of complexes containing human supervillin with one antibody and probing for supervillin in the precipitates by immunoblotting with the other.

The differences in efficiencies among these antibodies could have interesting underlying causes. For instance, the highly conserved Epitope 2 is very close to the sequence in supervillin that binds to the nonmuscle myosin II heavy chain [19]. Shielding of the epitope by binding to myosin II or the covalent modification of residues in this epitope by cytosolic enzymes or a chemical fixative could decrease the ability of the 4A8 and 5A8 antibodies to recognize their epitope(s). Alternatively, the conformationally dependent 4A8 and 5A8 epitopes might exist only in supervillin molecules that are bound to myosin II or to one of the other supervillin N-terminal interactors [18–23, 41]. This region of supervillin is part of a large intrinsically disordered protein sequence [49], sequences characteristic of signaling proteins capable of multiple conformations that are often regulated by their binding partners [50–54]. Differential epitope accessibility also could explain the incomplete circumferential staining observed around the periphery of muscle fibers stained with the 5A8 antibody (Fig 10), as compared with the previously observed complete circumferential staining observed with affinity-purified rabbit anti-h340 antibody [31]. Importantly, colocalization with dystrophin at costameres is visualized with both 5A8 (Fig 10E) and anti-H340 [31].

In conclusion, the antibodies characterized here should meet the needs of most investigations involving supervillin isoforms in mammalian cells and tissues. The 1E2 and 5A8 clones, in particular, hold great promise for the investigation of supervillin isoforms, localizations and functions during tumorigenesis and normal cell functioning. Each of the four antibodies extensively characterized here can be reproducibly generated in large quantities from hybridomas or by recombinant expression, and each will be available to the research community through the Clinical Proteomic Tumor Analysis Consortium’s Antibody Portal (antibodies.cancer.gov) and the Developmental Hybridoma Studies Bank (dshb.biology.uiowa.edu).

## Supporting information

S1 Fig Amino acid sequence alignments of the light and heavy chain variable regions of the sequenced antibodies against supervillin. Clones 1E2, 4A3, 2G3, 4A8 and 5B8 fall into two groups. Group 1 contains 1E2, 2G3 and 4A3; Group 2 consists of 4A8 and 5B8.
(DOCX)
