# Supplementary material for: Generation and characterization of monoclonal antibodies that recognize human and murine supervillin protein isoforms
Source: PLoS One. 2018 Oct 17;13(10):e0205910. doi: 10.1371/journal.pone.0205910 (PMC6192639; doi:10.1371/journal.pone.0205910)
Supplement: S1 Fig — Clones 1E2, 4A3, 2G3, 4A8 and 5B8 fall into two groups. Group 1 contains 1E2, 2G3 and 4A3; Group 2 consists of 4A8 and 5B8. (DOCX) [file pone.0205910.s001.docx]

**S1 Figure**

**Multiple Sequence Alignments of 1E2, 4A3, 2G3, 4A8, 5B8**

**Variable Light and Heavy Chain Amino Acid Sequences**

**Group 1. Variable Heavy Chain Regions**

4A3_HC QIQLQQSGPELVKPGASVKISCKASGYTFTDYYINWVKQRPGQGLEWIGWIYSGSGNTKY

2G3_HC QIQLQQSGPELVKPGASVKISCKASAYTFTDYYINWVKQRPGQGLEWIGWIYPGSGNTKY

1E2_HC QIQLQQSGPDLVKPGASVKISCKASGYTFTDYYINWVKQRPGQGLEWIGWIYPGSGNTKF

*********:***************.**************************.******:

4A3_HC NEKVKGEATLTVDTSSTTAYMQLSSLTSEDSAVYFCARGPFGGFAYWGQGTLVTVSA

2G3_HC NEKFKGKATLTVDTSSSTAYMQLSSLTSEDSAVYFCATGPFGGFAYWGQGTLVTVSA

1E2_HC NERFKDKATLTVDTSSSTAYMQLSSLTSEDSAVYFCASGPFGGFAHWGQGTLVTVSA

**:.*.:*********:******************** *******:***********

**Group 1. Variable Light Chain Regions**

1E2_LC DIVMTQAAPSVPVTPGESVSISCRSSKSLLYS-NGNTYLYWFLQRPGQSPQLLIYRMSNL

2G3_LC DIVMTQAAPSVPVTPGESVSISCRSSKSLLHS-NGNTYLYWFLQRPGQSPQLLIYRMSNL

4A3_LC DIVMSQSPSSLAVSVGEKVTMSCKSSQSLLYSSNQKKYLAWYQQKPGQSPKLLIYWAYTR

****:*:..*:.*: **.*::**:**:***:* * :.** *: *:*****:**** .

1E2_LC ASGVPDRFSGSGSGTAFTLKISRVEAEDVGVYYCMQHLEYPLTFGAGTKLELK

2G3_LC ASGVPDRFSGSGSGTAFALRISRVEAEDVGVYYCMQHLEYPLTFDAGTRLELK

4A3_LC ESGVPDRFTGSGSGTDFTLTISSVKAEDLAVYYCQQYYSYPFTFGSGTKLEIK

*******:****** *:* ** *:***:.**** *: .**:**.:**:**:*

**Group 2. Variable Heavy Chain Regions**

4A8_HC EVQLQQSGAELVRPGASVKLSCTASGFNIKDDHMHWVKQRPEQGLEWIGWIDPENGDTEY

5B8_HC QVQLQQSGAELVRPGASVTLSCKASGYTFTDYEMHWVKQTPVHGLEWIGAIDPETGGTAY

:*****************.***.***:.:.* .****** * :****** ****.*.* *

4A8_HC ASKFQDKATITADTSSNTAYLQFSSLTSEDTAVYYCTTG---------EFDNWGQGTTLT

5B8_HC NQKFKGKAILTADKSSSTAYMELRSLTSEDSAVYYCTRSGIYDGNYWWYFDVWDTGTTVT

.**:.** :***.**.***::: ******:****** . ** *. ***:*

4A8_HC VSS

5B8_HC VSS

***

**Group 2. Variable Light Chain Regions**

4A8_LC DIVMTQAAPSIPVTPGQSVSISCRSSKSLLHSNGNTYLYWFLQRPGQSPQLLIYRMSNLA

5B8_LC DIVMTQSHKFMSTSVGDRVSITCKAS-----QDVSTAVAWYRQKPGQSPKLLIYSASYRY

******: :..: *: ***:*::* .: .* : *: *:*****:**** *

4A8_LC SGVPDRFSGSGSATAFTLRISRVEAEDVGVYYCMQHLEYPFSFGAGTKLELK

5B8_LC TGVPDRFTGSGSGTDFTFTISSVQAEDLAVYYCQQHYSAPLTFGAGTKLELK

:******:****.* **: ** *:***:.**** ** . *::**********
